# Diacylglycerol Kinases: Regulated Controllers of T Cell Activation, Function, and Development

**DOI:** 10.3390/ijms14046649

**Published:** 2013-03-26

**Authors:** Rohan P. Joshi, Gary A. Koretzky

**Affiliations:** 1 Abramson Family Cancer Research Institute, University of Pennsylvania, Philadelphia, PA 19104, USA; E-Mail: rjoshi@mail.med.upenn.edu; 2 Department of Medicine, Perelman School of Medicine at the University of Pennsylvania, Philadelphia, PA 19104, USA

**Keywords:** diacylglycerol kinase, DGK, signal transduction, T cells, iNKT cells, anergy, tumor immunity

## Abstract

Diacylglycerol kinases (DGKs) are a diverse family of enzymes that catalyze the conversion of diacylglycerol (DAG), a crucial second messenger of receptor-mediated signaling, to phosphatidic acid (PA). Both DAG and PA are bioactive molecules that regulate a wide set of intracellular signaling proteins involved in innate and adaptive immunity. Clear evidence points to a critical role for DGKs in modulating T cell activation, function, and development. More recently, studies have elucidated factors that control DGK function, suggesting an added complexity to how DGKs act during signaling. This review summarizes the available knowledge of the function and regulation of DGK isoforms in signal transduction with a particular focus on T lymphocytes.

## 1. Introduction

Receptor-mediated signaling is critical for immune cell development and function. Engagement of the T cell receptor (TCR) on T cells activates signaling cascades that lead to their differentiation, proliferation, and elaboration of cytokines, which are required for optimal immunity. After engagement of the TCR by peptides displayed on antigen presenting cells (APCs), signaling is amplified by the activation of phospholipase C γ1 (PLCγ1), which cleaves phosphatidylinositol-4,5-biphosphate (PIP_2_) to form the second messengers diacylglycerol (DAG) and inositol triphosphate (IP_3_). Synthesis of DAG is crucial for activation of diverse downstream signaling cascades, including the Ras, NF-κB, and AKT pathways. DAG levels must therefore be finely tuned not only through controlled production but also by its metabolism. Diacylglycerol kinases (DGKs) are a diverse family of enzymes that phosphorylate DAG to form phosphatidic acid (PA), thereby terminating DAG signaling and initiating additional signaling events through the synthesis of bioactive PA. The critical role of DGKs in controlling DAG in T cells has become evident over the last decade through the use of both cell line and *in vivo* models. The regulation of DGKs themselves, however, has begun to be understood only recently. This review summarizes our understanding of the role of DGKs in T cells and describes new advances in deciphering the means by which DGKs are regulated.

## 2. DGK Isotypes and Their Structures

DGKs are a large family of enzymes that share a common substrate, DAG. The domain architectures of DGKs reflect this common function, as each of the ten isoforms has a catalytic domain as well as at least two C1 domains that are homologous to protein kinase C (PKC) C1 phorbol-ester/DAG binding domains.

The catalytic domains of DGKs contain two parts, a catalytic subdomain (termed DAGKc) and an accessory subdomain (termed DAGKa). The DAGKc subdomain contains a highly conserved Gly-Gly-Asp-Gly motif that serves as the ATP binding site and is necessary for enzymatic activity [1,2]. The role of the DAGKa subdomain has not been directly studied. However, two unrelated kinases, sphingosine kinase (SPK) and ceramide kinase (CERK), contain a domain similar to DAGKc, but do not contain a DAGKa subdomain. DAG is not a substrate for SPK and CERK, and DGKs are not specific for sphingosine or ceramide containing lipids, suggesting that the DAGKa subdomain may be required for the specificity of DGKs for DAG lipids [3–5].

The conservation of the C1 domain across all DGK isoforms suggests that this domain is essential for DGK function. Nevertheless, the exact role of the DGK C1 domain is unclear. As DGK C1 domains are homologous to DAG/phorbol ester-binding PKC C1 domains, DGK C1 domains were presumed to mediate the binding of DGK to DAG. However, no DGK C1 domain contains the canonical phorbol-ester binding amino acid motif of the PKC C1 domain, with the exception of DGKβ and DGKγ C1 domains [6]. DGK C1 domains therefore may not be involved in direct DAG binding. Nevertheless, the C1 domain may have non-canonical roles in controlling DGK localization. For example, cellular exposure to phorbol myristate acetate (PMA) causes DGKγ to translocate to the plasma membrane [7]. Targeted disruption of the C1 domain of DGKζ or DGKθ prevents the translocation of these molecules to the membrane in response to G-protein coupled receptor (GPCR) activation [8,9]. The DGK C1 domain may also be involved in protein–protein interactions, as evidence suggests that DGKζ translocation may be controlled by interactions with β-arrestins, rather than interactions with DAG [10]. In addition, DGKζ directly interacts with Rac1 through its C1 domain [11]. The available evidence suggests that the C1 domain may have functions unique to each isoform and could be involved in protein–protein interactions in addition to protein–lipid interactions.

### 2.1. DGK Subtypes

**Figure 1:**
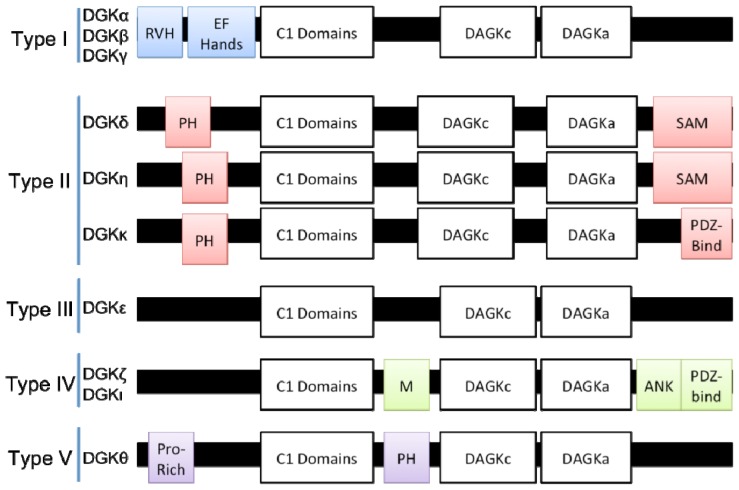
Diacylglycerol kinases (DGK) isoforms are divided into five subtypes based on domains apart from the C1 and catalytic domains. RVH: recoverin homology domain. PH: pleckstrin homology domain. SAM: sterile α motif. M: MARCKS homology domain. ANK, ankyrin repeat domain; PDZ-bind, PDZ-binding domain; Note, domain sizes are not to scale.

The ten DGKs are divided into five subtypes based on domains apart from the C1 and catalytic domains (Figure 1). As described below, these additional domains potentially regulate DGK catalytic activity, localization, and substrate specificity.

#### 2.1.1. Type I DGKs (DGKα, DGKβ, and DGKγ)

Type I DGKs contain an *N*-terminal calcium sensitive recoverin homology (RVH) domain and two EF hand motifs. The RVH domain is homologous to the *N*-terminus of the recoverin family of neuronal calcium receptors [12]. *In vitro* kinase assays using purified protein suggest that deletion of the RVH domain results in loss of calcium dependent activation of DGKα; in addition, simultaneous deletion of the RVH and EF hands domains results in constitutive activation of DGKα [12]. These data suggest that the RVH domain senses calcium, while the EF hand domain mediates suppression of kinase activity. The RVH and EF hands domains likely cooperate to control calcium-dependent activation of DGKα through an intramolecular interaction with the C1 and catalytic domains [13]. In addition to controlling enzymatic activity, binding of calcium to the EF hands may allow DGKα to translocate from the cytoplasm to the plasma membrane [14]. While the RVH and EF hand domains of DGKα are important for its function, the role of these domains in DGKβ and DGKγ function has been less well studied. When expressed in isolation, the EF hands of DGKβ and DGKγ bind to calcium with a dissociation constant an order of magnitude less than that of DGKα, suggesting that calcium may not strongly regulate these isoforms [15]. However, simultaneous deletion of the RVH and EF hands of DGKγ results in cytoplasm to plasma membrane translocation and increased filipodia-like cytoplasmic protrusions in a N1E-115 neuroblastoma cell line [16]. Thus, calcium is a key regulator of DGKα function and may regulate other Type I DGKs as well.

#### 2.1.2. Type II DGKs (DGKδ, DGKη, and DGKκ)

Type II DGKs contain an *N*-terminal plekstrin homology (PH) domain that mediates interaction with lipids and proteins [17–19]. The PH domain of DGKδ weakly binds to phosphatidylinositols; however, treatment of HEK293 cells with the DAG analog PMA promotes PH domain-dependent translocation of DGKδ to the plasma membrane [20–23]. In addition to the PH domain, DGKδ and DGKη contain a sterile α motif (SAM) at their carboxy termini, which may be involved in oligomerization through zinc binding [22,24]. DGKκ lacks a SAM domain but does contain a *C*-terminal motif that may bind type I PDZ domains [17]. The function of this motif is unknown.

#### 2.1.3. Type III DGKs (DGKɛ)

DGKɛ, the only member of the Type III DGK family, contains no additional structural domains other than its tandem C1 and catalytic domains. Interestingly, DGKɛ is the only DGK isoform known to display specificity to the acyl sidechains of the glycerol backbone of DAG [25,26]. DGKɛ preferentially binds to DAG containing an arachidonoyl chain at middle position of the glycerol backbone [25,26]. This preference may be responsible for the presence of arachidonoyl containing PIP_2_ in the plasma membrane [27,28].

#### 2.1.4. Type IV DGKs (DGKζ and DGKι)

Type IV DGKs contain myristoylated alanine-rich protein kinase C substrate (MARCKS), PDZ-binding, and ankyrin domains. The MARCKS domain is homologous to the phosphorylation-site domain (PSD) of the MARCKS protein and contains four serine residues that are possibly sites of phosphorylation by protein kinase C (PKC) α [2]. In COS-7 and A172 cells, this phosphorylation event leads to nuclear translocation of DGKζ; in Jurkat T cells, serine to alanine mutation of these residues results in loss of translocation of DGKζ to the plasma membrane [2,8]. The PDZ-binding and ankyrin domains are involved in protein–protein interactions. For example, the PDZ-binding domain regulates DGKζ’s interaction with Sorting Nexin-27 (SNX27) and is required for SNX27 to mediate intracellular vesicle trafficking [29]. Additionally, in Purkinje neurons and muscle cells, the PDZ-binding domain controls DGKζ’s interaction with γ1-Syntrophin, which determines the subcellular localization of DGKζ [30–32]. Finally, the ankyrin domain regulates DGKζ’s interaction with the long form of the leptin receptor in rat hypothalamus extract and may regulate leptin receptor signaling [33]. The role of the MARCKS and ankyrin domains in DGKι function is unknown. However, just as has been found for DGKζ, the PDZ-binding domain is required for the interaction of DGKι with the postsynaptic density 95 (PSD-95), a neuronal scaffolding protein [34,35].

#### 2.1.5. Type V DGKs (DGKθ)

The only member of the Type V DGK family, DGKθ, contains an *N*-terminal proline-rich domain, a PH domain between its C1 and catalytic domains, and a Ras-association domain within its PH domain. The enzymatic activity of DGKθ is diminished by mutations of the PH and proline-rich domains, suggesting an essential role of these species conserved regions in DGKθ function [36,37].

## 3. Tissue Expression of DGKs

DGK expression is broad, as most tissues express multiple DGK isoforms and as most DGK isoforms are expressed in multiple tissues. The major site of DGK expression is the brain, which expresses all known DGK isoforms [28,38]. A recent review reported expressed sequence tag (EST) data available from the National Center for Biotechnology Information (NCBI) and summarized tissue expression of DGKs [28]. Interestingly, EST data suggest that DGK isoform expression is relatively narrow in several tissues: (1) DGKɛ is the only expressed isoform in adipose tissue; (2) DGKγ is the only isoform expressed in the pituitary gland; (3) DGKα and DGKθ are the only isoforms expressed in the bone marrow; and (4) DGKα, DGKδ, and DGKζ are the most abundant isoforms expressed in lymphocyte-rich tissue [28]. While DGKα, DGKδ, and DGKζ are all expressed in T cells, the function of DGKδ in T cells remains unknown. As such, the role of DGKα and DGKζ in T cells is discussed in detail below.

## 4. Regulation of DGK Function in T Cells

### 4.1. Transcriptional Regulation of DGKs in T Cells

The regulation of DGKα and DGKζ has been a recent focus of research because of the key roles these molecules have in TCR signaling. Both DGKα and DGKζ are regulated at the transcriptional level. T cell lines that are induced to become unresponsive to antigen (anergic) by the administration of TCR stimulation without co-stimulation through additional receptors, such as CD28, up-regulate DGKα mRNA [39,40]. In contrast, activation of murine T cells with both TCR and co-stimulation causes down-regulation of both DGKα and DGKζ mRNA transcripts [41,42]. These data suggest that transcription of DGKα and DGKζ mRNA is sensitive to signals downstream of TCR and co-stimulation.

One transcription factor upregulated in response to TCR stimuli is early growth responsive gene 2 (Egr2). A binding site for Egr2 was identified within the promoter of DGKα [43]. After TCR engagement, transcription of Egr2 was found to peak before DGKα transcription increased, suggesting that Egr2 may regulate DGKα levels. It was found that Egr2 bound to the DGKα promoter and increased DGKα transcription after TCR engagement in the absence of co-stimulation. Furthermore, deletion of Egr2 resulted in decreased DGKα upregulation and anergy induction after anergy inducing conditions. In addition, forced over-expression of DGKα in Egr2-deficient T cells rescued anergy induction. These results suggest that Egr2 is a key regulator of DGKα transcription during anergy and that this regulation has important functional effects.

Recently, binding sites for members of the forkhead box O (FoxO) transcription factor family were also found within the DGKα gene [42]. In quiescent T cells, FoxO1 and FoxO3 are bound to the DGKα promoter and enhance DGKα transcription. DGKα promoter binding is lost after T cell activation, and this event is correlated with the phosphorylation of FoxO proteins downstream of TCR and AKT signaling and decreased DGKα transcription. T cells exposed to the cytokine interleukin-2 (IL-2), which is produced by activated T cells, were also found to decrease DGKα transcription in a manner dependent on AKT function. In addition, this decreased transcription correlated to decreased FoxO binding to the DGKα promoter. Hence, FoxO proteins also appear to provide a link between TCR signaling, T cell activation, and DGKα transcription. Thus, in quiescent cells, FoxO-mediated transcription of DGKα may be high, whereas initial TCR signaling may lead to decreased FoxO-mediated transcription and the production of IL-2, serving to further diminish DGKα transcription in activated T cells. Further studies using genetic tools would help determine the functional implication of FoxO regulation of DGKα on T cell activation and anergy. The factors that control DGKζ transcription have not yet been studied.

### 4.2. Post-Translational Regulation of DGKs in T Cells

Evidence also exists for post-translational regulation of DGKα and DGKζ. The presence of RVH and EF hand domains in DGKα suggests that cytoplasmic calcium levels could regulate this isoform. After TCR engagement, calcium is released into the cytoplasm from the ER and extracellular environment [44]. *In vitro* data would suggest that this flux would lead to binding of calcium to the EF hands and loss of auto-inhibition (*i.e.*, activation) of DGKα catalytic activity [12,13]. Surprisingly, treatment of Jurkat T cells with an intracellular calcium chelator during TCR and co-stimulation leads to an increase rather than a decrease in DGKα catalytic activity as measured by an *ex vivo* assay of PA production [45]. Interestingly, intracellular calcium increases are necessary for the translocation of DGKα to the plasma membrane, where DAG is synthesized after TCR engagement [1,46]. Thus, the *in vivo* calcium-dependent regulation of DGKα is more complex than what occurs *in vitro* with isolated protein, and calcium may serve to localize DGKα activity rather than merely modulate DGKα catalytic function.

Recently, the adapter protein SAP (signaling lymphocyte activation molecule (SLAM)-associated protein) was also found to regulate DGKα function during immune cell signaling [45]. Upon TCR and co-stimulation of Jurkat T cells through either CD28 or SLAM receptor engagement, DGKα is recruited to the membrane, and its catalytic activity is suppressed. This suppression is dependent upon PLCγ1 activation and calcium release [45]. Knockdown of SAP results in a rescue of DGKα catalytic activity and reduced translocation to the plasma membrane after TCR and co-stimulation [45]. The investigators in this study could not find a direct protein interaction between SAP and DGKα; thus, SAP apparently directs the posttranslational regulation of DGKα through an as yet unidentified mechanism [45]. The Src family tyrosine kinase Lck also regulates DGKα in a T cell line [47]. TCR stimulation of Jurkat T cells leads to Lck-mediated phosphorylation of DGKα at the hinge region between its tandem C1 domains and the catalytic domain [47]. Mutation of this tyrosine residue to phenylalanine abrogates DGKα translocation to the plasma membrane and suppression ERK phosphorylation [47]. TCR-activated Lck phosphorylation of DGKα therefore is an important event for attenuating T cell activation.

Comparatively little is known about posttranslational modification of DGKζ in immune cells. As described above, in COS-7 and A172 cells, PKCα phosphorylates serine residues in the MARCKS domain of DGKζ and leads to its nuclear translocation [2]. In Jurkat T cells, mutation of these serine residues to alanine results in loss of DGKζ translocation to the plasma membrane, but the kinase that acts on DGKζ has not been definitively identified [8,48]. Although the MARCKS domain appears important for localization in this cell line, the importance of this domain for DGKζ regulation of downstream signaling pathways requires further study.

## 5. DGK Regulation of Immune Cell Signaling and Activation

### 5.1. An Overview of TCR and DAG Signaling in T Cells

**Figure 2:**
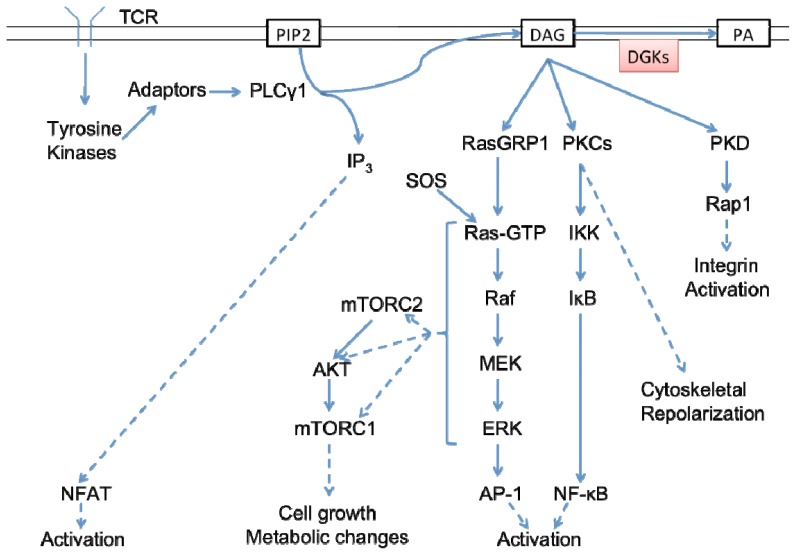
Diacylglycerol has a central role in TCR signaling. Engagement of the TCR leads to the activation of tyrosine kinases and the formation of a multimolecular complex including adapter proteins. PLCγ1 is recruited to and activated by this complex and in turn hydrolyses the phospholipid PIP_2_ to form membrane-diffusible diacylglycerol (DAG) and cytosolic IP_3_. DAG activates proteins including RasGRP1, PKCs, and PKD. Activation of RasGRP1 leads to Ras and AKT pathway activation. Activation of PKCs leads to the activation of NF-κB and cytoskeletal repolarization. Activation of PKD leads to integrin activation. IP_3,_ Ras, and PKC-θ signaling cooperate to promote T cell activation through the transcription factors NFAT, AP-1, and NF-κB, respectively. Diacylglycerol kinases (DGKs) phosphorylate DAG to form phosphatidic acid (PA), thereby potentially regulating a broad set of signaling pathways downstream of the TCR.

Engagement of the TCR by peptide presented by APCs starts a signaling cascade that culminates in activation of the T cell. Early events lead to the activation of the tyrosine kinases Lck and ZAP-70, which leads to the formation of a multimolecular signaling complex involving the adapter proteins linker of activated T cells (LAT) and SH2 domain containing leukocyte protein of 76 kDa (SLP-76) [44,49,50]. PLCγ1 is recruited to and activated by this complex and in turn hydrolyses the phospholipid PIP_2_ to form membrane-diffusible DAG and cytosolic IP_3_[51] (Figure 2). DAG recruits and activates numerous targets, of which the best characterized in T cells are RasGRP1, PKD, and PKCθ, although other PKCs are targets as well [52–54]. RasGRP1 is a guanine nucleotide exchange factor that activates Ras-GTP and the MAPK cascade, along with non-DAG activated son of sevenless (SOS) [55]. This leads to the formation of the activator protein 1 (AP-1) transcription factor complex, which is critical for the production of IL-2 and T cell activation. Activation of PKCθ leads to the recruitment of CARMA1 and Bcl10, culminating in the activation of the NF-κB pathway, which is important for the elaboration of pro-inflammatory cytokines [56–61]. Activation of PKD promotes integrin activation through an interaction with Rap1 in T cells [62]. DGKs phosphorylate DAG to form PA and may act as negative regulators of these pathways.

PA itself is an important signaling lipid, although its functions in T cells are not well defined. Data from a number of systems suggest that the role of PA in T cell activation is complex, as the source of PA and its localization within the cell may determine how this second messenger functions. For example, in Jurkat T cells, simultaneous activation of integrin and TCR receptors leads to hydrolysis of phosphatidylcholine by phospholipase D 2 (PLD2), likely creating a pool of PA that is a source for the synthesis of DAG by phosphatidic acid phosphatase (PAP) [63]. This PLD2-sourced pool of DAG is important for specific activation of k-Ras, rather than n-Ras, which is activated by TCR signaling alone [63]. PA may also lead to activation of mammalian target of rapamycin (mTOR), and DGKζ specifically may influence this pathway [64–67]. For example, in HEK293 cells, overexpression of DGKζ increased PA levels in response to mitogenic simuli. Overexpression of DGKζ also increased phosphorylation of ribosomal S6 kinase (S6K), a molecule downstream of mTOR, in a manner dependent on mTOR’s PA binding domain [67]. These data suggest that, after engagement of the TCR, DAG and DGKs may have additional roles in regulating mTOR and other pathways downstream of PA. As discussed below and consistent with this notion, PA may have a role in the development of T cells in the thymus. Further studies are needed, however, in primary T cells to further elucidate the functions of PA in T cells.

### 5.2. DGKα in T Cell Signaling and Activation

DGKs serve a crucial role in T cells by metabolizing DAG and terminating DAG-activated signaling cascades. The predominant isoforms of DGK expressed in T cells are DGKα and DGKζ [68]. Studies in a transformed T cell line (Jurkat) stimulated through an ectopically expressed muscarinic type I receptor revealed a modest reduction in CD69 upregulation with overexpression of wildtype DGKα and a marked reduction of CD69 upregulation with expression of a constitutively active form of DGKα [1]. Overexpression of DGKα in Jurkat T cells also reduced AP-1 activation after TCR stimulation [41]. In studies of a virally transduced primary T helper 1 cell clones, overexpression of DGKα was found to inhibit ERK phosphorylation in response to TCR and co-stimulation and translocation of RasGRP1 to the plasma membrane [39]. Primary T cells deficient in DGKα also demonstrate increased ERK phosphorylation and Ras activation following TCR stimulation [41]. This leads to increased IL-2 production and proliferation of DGKα-deficient cells during blockade of co-stimulatory signals [41]. Together, these data suggest that DGKα-dependent suppression of TCR signaling is crucial for the suppression of T cell activation. The effects of DGKα deficiency on primary T cell function are discussed further below.

### 5.3. DGKζ in T Cell Signaling and Activation

Early experiments in which DGKζ was overexpressed in Jurkat T cells demonstrated that DGKζ suppresses Ras, ERK, and AP-1 activation as well as upregulation of the activation marker CD69 [69]. Kinase activity was found to be required for this suppression; in addition, an intact *N*-terminal region including the MARCKS domain was required, while the ankyrin and PDZ-binding domains were found to be dispensable [69]. Subsequent experiments in primary T cells from DGKζ-deficient mice confirmed these findings. DGKζ knockout T cells produced less PA after TCR stimulation [70]. In addition, phosphorylation of MEK and ERK was increased in DGKζ-deficient T cells. This increase correlated with increased upregulation of the activation markers CD69 and CD25 as well as increased proliferation to antigen and increased homeostatic proliferation (expansion of cell numbers when transferred into lymphopenic mice) [70]. Finally, DGKζ-deficient mice had a modest increase in responses to the pathogen lymphocytic choriomeningitis virus (LCMV), indicating that enhanced T cell signaling and activation may have functional consequences as well (discussed further below).

### 5.4. DGK Regulation of AKT-mTOR Signaling in T Cells

Much evidence suggests, therefore, that DGKα and DGKζ have overlapping roles in T cell signaling, although no direct comparison of the relative contribution of DGKα and DGKζ to this regulation has been performed. Highlighting the overlapping yet complementary roles of DGKα and DGKζ, treatment of DGKζ-deficient T cells with an inhibitor of DGKα function increases IL-2 production and proliferation in response to TCR stimuli and co-stimulation blockade compared to DGKζ-deficient untreated cells [41]. In addition, simultaneous deletion of DGKα and DGKζ in T cells [double knockout (DKO) cells] increases activation of the AKT and mTOR pathways [71]. Increased Ras-MEK-ERK-Rsk1 signaling in DKO cells is likely responsible for increased AKT-mTOR activation [71]. DGKα and DGKζ are therefore responsible for regulating a wide range of pathways that are important for T cell signaling, activation, proliferation, and metabolism.

### 5.5. DGKζ as a Modulator of Analog-to-Digital Signaling in T Cells

The role of DGKζ in T cell activation has been refined recently with respect to Ras activation. T cells exhibit digital activation after TCR stimulation, such that increases in TCR ligand binding lead to increased percentages of cells with an “on” state, rather than a smooth gradient of cell activation markers [72]. This mechanism requires that an analog signal (number of TCR bound to ligand) be converted to a digital state (activation) through the TCR signaling machinery. The Ras pathway contributes to this conversion through the two guanine nucleotide exchange factors RasGRP1 and SOS [73]. DAG allosterically activates RasGRP1; in contrast, the immediate product of RasGRP1 and SOS–Ras-GTP–allosterically activates SOS, thereby creating a positive feedback loop [73,74]. In other words, as Ras-GTP production by RasGRP1 reaches a certain threshold, SOS is activated and greatly enhances production of Ras-GTP. Thus, DAG-RasGRP1 units serve as an analog component, while SOS serves as an analog to digital converter [72,75]. As DGKζ activity attenuates DAG, it is positioned to regulate the number of DAG-RasGRP1 molecules present and therefore modulate the threshold for digital Ras activation. This phenomenon was studied in CD8^+^ T cells [76]. An *in silico* model predicted that CD8^+^ T cells deficient in DGKζ would have increased DAG-RasGRP1 functional units, leading to an increase in fraction of T cells with activated Ras signaling but no change in the amount of activated Ras in each cell. *In vitro* testing using phosphorylation of ERK as a surrogate for Ras activation confirmed this prediction. In addition, the model predicted that a lower amount of antigen-receptor engagement on DGKζ-deficient cells than wildtype cells would be necessary for the equivalent amount of Ras/ERK activation. However, *in vitro* testing of this notion found that wildtype and DGKζ-deficient CD8^+^ T cells had similar levels of antigen-receptor engagement at 50% of the maximum ERK phosphorylation, suggesting increased complexity to how DGKζ controls RasGRP1 activity. These data therefore suggest that DGKζ activity is important for determining the threshold of T cell activation. Additional studies are needed to determine whether the presence of DGKα in T cells also affects this threshold.

### 5.6. DGK at the Immunological Synapse

The immunological synapse (IS) forms at the site where the TCR engages with antigen presented by an APC. A multimolecular signaling cluster is formed at this site, which includes PLCγ1. The localized production of DAG at the IS is important for functional TCR signaling. For example, localized DAG synthesis causes reorientation of the microtubule-organizing complex (MTOC) towards the IS, which is required for transduction of the TCR signal [77]. Moreover, treatment of TCR-stimulated cells with an inhibitor of DGK activity results in diffuse DAG persistence at the IS and impaired MTOC reorientation, which is thought to be important for directional secretion of cytolytic factors and sustained TCR signaling [53,77]. DGKs may therefore play an essential role at the IS in metabolizing DAG and restricting DAG localization to the IS. This notion was studied in Jurkat T cells [48]. Following TCR stimulation, TCR-bound immune complexes were isolated, and both DGKα and DGKζ were present, although a GFP-tagged DGKα did not translocate to the plasma membrane as visualized by microscopy. In addition, knockdown of DGKα did not affect the *in vitro* conversion of DAG to PA by the TCR-bound complexes, while knockdown of DGKζ did. Collectively, these experiments support the notion that while both DGKα and DGKζ likely participate in the regulation of DAG at the IS, DGKζ appears to play the dominant role. It was also found that treatment of Jurkat T cells with PMA enhanced the accumulation of DGKζ in TCR-bound immune complexes, suggesting a positive feedback between DAG accumulation and DGKζ function at the IS [48]. Although it was not formally shown, this could be due to stimulation of PKCs, as a non-phosphorylatable MARCKS domain mutant DGKζ was defective in DAG metabolism at the IS. Together, these data suggest a key role for DGKs, in particular DGKζ, in regulating signaling at the IS.

DGKs may also be involved in secretory traffic at the IS. One mechanism of target cell killing at the IS by CD8^+^ cytotoxic T lymphocytes (CTLs) may be the engagement of Fas on target cells by Fas ligand (FasL) expressed on the surface of CTLs or by FasL secreted through exosomes [78]. Exosomes containing FasL form within multivesicular bodies (MVBs) in CTLs and may be regulated by T cell activation [78,79]. In a recent study, Jurkat T cells were used as a model system to study the role of DGKα in MVB exosome secretion [80]. DGKα was recruited to MVBs and appeared to have two distinct roles. Through pharmacologic inhibition, it was shown that DGKα activity negatively regulated mature MVB formation. In contrast, knockdown of DGKα resulted in increased polarization of MVBs to the IS and increased exosome secretion. These results are consistent with the notion that DGKα has a dual role in exosome secretion, with kinase activity required for suppression of MVB development but non-kinase functions required for exosome secretion. The role of DGKζ in MVB formation requires further study.

## 6. DGK Functions in T Cells

### 6.1. T Cell Anergy

Anergy is one mechanism by which T cells undergo peripheral tolerance. Engagement of the both the TCR as well as co-stimulatory receptors leads to normal T cell activation, whereas stimulation of T cells through the TCR alone induces anergy [40,81,82]. Anergic cells do not proliferate or produce IL-2 in response to antigenic stimuli, thus preventing the potential activation of self-reactive cells that have escaped negative selection in the thymus [82]. Anergic T cells were found to have decreased Ras activation and translocation of RasGRP1 to the immunological synapse, although activation of nuclear factor of activated T cells (NFAT), a calcium-dependent process has been found to be intact [39,83–85]. Thus, it appears that anergy correlates with a normal TCR-induced calcium signal but defective production of DAG. As IP_3_ and DAG are created in equimolar ratios by PLCγ1 cleavage of PIP_2_, it was speculated that DGK metabolism of DAG could potentially alter the balance of calcium and DAG signaling, thus converting T cell activation into T cell anergy. This notion was lent further weight by microarray data in which DGKα was found to be one of the predominant transcripts upregulated in anergic T cells [39,40].

Experimental evidence from studies in which DGK levels were manipulated genetically support the role of DGKs in T cell anergy. Overexpression of DGKα in a T cell line mimics an anergic state characterized by decreased Ras activation and RasGRP1 translocation to the IS [39]. Conversely, deletion of DGKα or DGKζ in primary cells results in cellular resistance to anergy [41]. Engagement of cytotoxic T lymphocyte antigen 4 (CTLA4), an inhibitory receptor on the surface of T cells, in the presence of TCR stimulation normally suppresses activation and promotes anergy. However, unlike wildtype cells, stimulation of DGKα- or DGKζ-deficient T cells through the TCR in the presence of co-stimulation blockade with CTLA4-Fc resulted in proliferation and production of IL-2 [41]. Moreover, using an *in vivo* model, DGKα-deficient mice were found to have impaired anergy induction [41]. Together, these data demonstrate that DGKα and DGKζ both function to control T cell activation and perhaps aid peripheral tolerance.

Neither DGKα- nor DGKζ-deficient mice develop autoimmunity, which may be expected for molecules that suppress T cell activation. Autoimmune hepatitis is observed in mice that lack both isoforms, suggesting that DGKα and DGKζ have redundant functions in suppressing autoimmunity [68]. However, the DKO mice have striking defects in T cell selection and development (discussed below). Hence, although T cells deficient in both isoforms demonstrate augmented TCR activation and potentially a propensity for inducing autoimmunity, another possibility is that these T cells express an altered set of TCRs that could potentially lead to an autoimmune phenotype. Separating these possibilities is difficult in conventional DKO mice, as normal T cell development does not occur. Mice with conditional gene targeting systems would help clarify the mechanisms of autoimmunity in DGK-deleted mice, as these mice would enable normal T cell development to proceed before gene deletion of DGKα and DGKζ. These mice would also allow investigators to separate possible alterations in selection from changes in TCR signaling in mature T cells for the observed effects of DGK loss on T cell activation and anergy, described above.

### 6.2. T Cell Responses to Pathogen

Effective immune responses to initial exposure to a pathogen as well as subsequent exposures are essential for survival of the host. Effector and memory T cell subsets mediate these responses, respectively. TCR signal strength modulates both effector and memory T cell differentiation and maintenance [86–89]. The critical role of DGKα and DGKζ in suppressing T cell activation and the regulated expression of these molecules during activation led to a study of how these molecules function in effector and memory responses to LCMV [90]. CD8^+^ T cells from LCMV-specific P14 transgenic TCR mice deficient in DGKα or DGKζ showed increased antigen-specific CD8^+^ T cell expansion and increased cytokine production in response to LCMV infection. Interestingly, the enhanced effector responses to LCMV by DGKα-deficient T cells were found to be partly cell extrinsic, while the enhanced responses to LCMV by DGKζ-deficient T cells were found to be cell intrinsic; these data suggest separate roles for DGKα or DGKζ in T cell signaling. After rechallenge with LCMV, CD8^+^ T cells deficient in DGKα or DGKζ demonstrated decreased expansion, indicating that DAG signaling may differentially control effector and memory cell formation.

The mechanism controlling this differential DGK function in effector and memory T cells is currently unclear. One intriguing possibility is that DAG signaling may control mTOR activation, which acts differently in effector and memory T cells [90–93]. Inhibition of mTOR complex 1 (mTORC1) function using rapamycin has been found to diminish CD8^+^ effector T cell function and promote memory CD8^+^ T cell differentiation [93,94]. These data indicate that one function of mTORC1 is to enhance effector T cell function and diminish memory T cell formation, which mirrors the observed phenotype in mice deficient in DGKα or DGKζ. As described above, T cells lacking both DGKα and DGKζ have increased AKT and mTORC1 activation [71]. In addition, although we have not investigated mTORC1 activation, we have found that T cells lacking DGKζ alone have increased AKT activation (unpublished observations). Taken together, the available evidence suggests that deletion of DGKs may regulate effector and memory responses by enhancing mTORC1 activity, although further studies are required.

### 6.3. T Cell Anti-Tumor Responses

T cell responses to tumor are often characterized by development of peripheral tolerance to tumor antigens [95]. Induction of T cell anergy in particular may be one mechanism by which tumors evade the immune system [95]. As deletion of DGKα or DGKζ disrupts anergy formation and promotes T cell activation, deletion of these molecules may also enhance T cell responses to tumor. Other inhibitory molecules involved in TCR signaling have been targeted successfully to increase T cell responses to tumor. For example, CD8^+^ T cells deficient in SH2 domain-containing phosphatase 1 (SHP-1), a tyrosine phosphatase, or cbl-b, an E3 ubiquitin ligase, have enhanced responses to tumor. Recently, the role of DGKζ in tumor responses was tested using an implanted EL-4 OVA model of tumor eradication [76]. Compared to tumors implanted in wildtype mice, tumors implanted into DGKζ-deficient mice were significantly smaller after three weeks. In addition, tumor antigen-specific T cells from DGKζ-deficient mice expressed higher levels of markers of activation and proliferation. To rule out the effects of DGKζ deletion in non-T cells and determine whether CD8^+^ T cells were responsible for this effect, these experiments were also performed using adoptively transferred CD8^+^ DGKζ-deficient T cells, with similar results.

DGKζ-deficient T cells did not display enhanced cytotoxicity compared to wildtype CD8^+^ T cells. The mechanism by which DGKζ deficiency enhances tumor responses is an important question and requires further investigation. For example, DGKζ deficiency could enhance anti-tumor responses primarily by preventing the induction of anergy during initiation of the immune response. Alternatively, DGKζ deficiency may increase anti-tumor responses by lowering the threshold for T cell activation during proliferation of effector cells. Distinguishing between these possibilities will have important implications for the timing of administration of DGKζ-targeting cancer immunotherapies. DGKζ has added promise as a therapeutic target compared to other inhibitory molecules, as unlike mice deficient in SHP-1 or cbl-b, mice lacking DGKζ do not develop overt signs of autoimmunity. Additional study is needed, however, to determine if modulation of DGKζ function is also applicable to T cell responses in non-implanted, endogenously developing tumor models.

The role of DGKα in anti-tumor responses was studied recently in human tumor-infiltrating CD8^+^ T cells (CD8-TILs) from patients with renal cell carcinoma (RCC) [96]. CD8-TILs from RCCs were defective in lytic granule exocytosis and their ability to kill target cells. While proximal signaling events were intact in response to TCR engagement, CD8-TILs exhibited decreased phosphorylation of ERK when compared to non-tumor-infiltrating CD8^+^ T cells. This impairment of lytic ability and phosphorylation of ERK correlated with an increase in DGKα expression in CD8-TILs. Treatment of CD8-TILs with an inhibitor of DGKα activity rescued killing ability of target cells, increased basal levels of phosphorylation of ERK, and increased PMA/ionomycin-stimulated phosphorylation of ERK. In addition, *ex vivo* administration of IL-2 to CD8-TILs was found to decrease DGKα expression levels, rescue lytic granule exocytosis and killing ability, and increase phosphorylation of ERK in response to PMA/ionomycin treatment. These results suggest that CD8-TILs may be defective in function partially due to high DGKα levels, leading to dysfunction in lytic granule exocytosis, which is consistent with the previously described role for DGKα in controlling secretory traffic at the immunological synapse [80]. Further study is needed to elucidate the DGKα-regulated pathways in CD8-TILs that control lytic granule exocytosis.

### 6.4. T Cell Adhesion

Surveillance of a host for pathogens requires that T cells constantly circulate between secondary lymphoid organs and enter and exit microvasculature. Interaction with the microvasculature requires cooperation between (1) selectins, which mediate rolling on endothelial cells, (2) chemokine receptors, which determine directionality of cellular migration and transduce signals to activate integrins on the migrating cell, and (3) integrins, which mediate the firm attachment of the cell to the underlying endothelium and allow transmigration into a tissue [97,98]. DAG-activated RasGRP2 may link chemokine receptor signaling to integrin activation by activating Rap1 [97]. Through DAG metabolism, DGKs are therefore positioned to regulate signaling from chemokine receptors to integrins, so-called “inside out” signaling. The role of DGKζ in regulating T cell adhesion was recently studied [99]. Using a quantitative model of T cell integrin signaling adhesive dynamics, it was predicted that DGKζ deficiency would enhance the kinetics of integrin activation and shorten the distance to arrest of T cells under shear flow [99]. To test this model, wildtype or DGKζ-deficient T cells were introduced into a flow chamber with immobilized P-selectin and intercellular adhesion molecule-1 (ICAM-1), a ligand for integrins, with or without the chemokine receptor ligand CXCL12. The time and distance to arrest of T cells of the different genotypes was then measured. DGKζ-deficient T cells had a significantly shorter time and distance before arrest than wildtype cells after addition of CXCL12, supporting the model and indicating that DGKζ deficiency may enhance integrin activation kinetics and firm arrest of T cells. These data suggest that DGKζ regulates T cell arrest and may suppress aberrant integrin activation. Further studies are required to investigate the molecular mechanisms by which DGKζ modulates inside-out signaling and integrin activation.

## 7. DGKs and Development

### 7.1. T Cell Development

During T cell development in the thymus, each thymocyte expresses a TCR with a single specificity. These TCRs arise from the random rearrangement of gene segments resulting in enormous potential for diversity of sequence. The selection of thymocytes from this large number of developing cells with useful but self-tolerant TCRs is essential for a productive immune response to pathogens that does not cause autoimmunity. Signaling through the TCR of developing T cells directs the deletion of cells that cannot recognize peptide presented by self-MHC (termed positive selection) and cells that are potentially autoreactive (termed negative selection). The TCR-mediated, DAG-activated RasGRP1 is a critical molecule for the positive selection of T cells [55,100]. In addition, Ras/ERK signaling is important for both positive and negative selection of T cells [101–103]. Strong but transient ERK signals induce negative selection, whereas weaker but sustained ERK signals promote positive selection [101–103]. Recruitment of activated Ras to the plasma membrane may lead to strong selecting signals, while recruitment to both the plasma membrane and intracellular regions may lead to weak selecting signals [101]. Together, these data suggest that alterations in DAG signaling by DGKs could alter thymocyte selection by altering strength of signal and Ras-GTP localization.

Expression of both DGKα and DGKζ is highly regulated during thymocyte development. DGKα mRNA is expressed at low levels in the earliest double negative (DN) stage of development and increases as thymocyte development progresses to the double positive (DP) and CD4 and CD8 single positive (CD4SP and CD8SP) stages [104–107]. Two isoforms of DGKζ are expressed in developing thymocytes, a short form and long form that share the same domains but are distinguished by the length of the *N*-terminal region [69]. The long isoform is more highly expressed than the short isoform in DN thymocytes; as development continues, expression of the short isoform progressively increases while expression of the long isoform decreases [69]. Whether this switch in isoform has functional implications is unknown.

While both molecules demonstrate regulated expression during development, deficiency of DGKα or DGKζ alone does not grossly alter thymocyte selection, although we have observed a decrease in CD8SP thymocytes in mice deficient in DGKζ [41,70,108]. DGKα and DGKζ may cooperate to control selection, as simultaneous deletion of DGKα and DGKζ leads to a strong block at the DP stage of thymocyte development [109]. Interestingly, upregulation of CD69, CD5, and TCRβ—events correlated with productive positive selection—are unaffected, and this defect is likely due to increased cell death once thymocytes reach the TCRβ^hi^ stage of DP development [109]. Positive selection can be tested using female mice that have a transgenic TCR specific for the H-Y antigen, which is only expressed by males. Female DKO mice crossed to the H-Y transgene have decreased CD8SP compared to wildtype, but whether this is due to a failure of positive selection or to enhanced negative selection is unclear. Mice with RasGRP1 overexpression do not have defects in positive selection, suggesting that DGK deficiency may lead to alterations in signaling through molecules other than RasGRP1 [109,110]. Although further study is needed, exogenous addition of PA to DGKα- and DGKζ-deleted fetal thymic organ cultures partially rescued development of SP thymocytes, indicating that lack of PA production during TCR signaling could lead to the observed phenotype [109]. In summary, these data provide strong evidence that DGKα and DGKζ have a cooperative role in controlling the selection of developing thymocytes.

### 7.2. Invariant NKT Cell Development

Invariant natural killer T cells (*i*NKT) are a rare subset of T cells that function at the interface of innate and adaptive immunity. Rather than expressing a wide range of TCRs, *i*NKT cells generally express a restricted set of TCRs that are stimulated by lipid antigens presented by gene products of the CD1 locus, molecules that are similar to classical major histocompatibility proteins. *i*NKT cells are thought to be involved in immune responses to tumors and a subset of pathogens. Their dysregulation appears to be important in the development of autoimmunity, diabetes, and asthma [111]. After TCR rearrangement, *i*NKT cells progress through the CD4^+^CD8^+^CD24^hi^ stage of T cell development, in which they undergo selection on CD1d-expressing cortical thymic epithelial cells [112,113]. After selection, *i*NKT cells downregulate CD24 and continue to mature, upregulating CD44 and finally NK1.1 [111]. Proper *i*NKT cell development in the thymus requires homotypic interaction of SLAM family receptors as well as their associated signaling kinases Fyn and SAP [114–118]. In addition, the PKCθ/NF-κB pathway plays a critical role in *i*NKT cell development at multiple stages [111]. As DAG activates PKCs, a recent study investigated the role of DGKα and DGKζ in *i*NKT cell development [119]. Although deficiency of DGKα and DGKζ alone does not markedly affect *i*NKT cell development, simultaneous deletion of both isoforms reveals a cell-intrinsic defect in *i*NKT cell development at the early CD44^lo^NK1.1^−^ stage. After TCR stimulation, thymocytes lacking both DGKα and DGKζ had increased ERK and IκBα phosphorylation compared to wildtype cells, suggesting that DGKs may enhance *i*NKT cell development through suppression of these pathways. Consistent with this notion, mice with constitutively active IKKβ had *i*NKT cell development that resembled that of DGKα and DGKζ double deficient mice, although there was not a correlation when all surface markers were investigated. In contrast, early *i*NKT cell development was unaffected in mice with constitutively active k-Ras; instead, these mice displayed a severe block at the transition from CD44^hi^NK1.1^−^ to CD44^hi^NK1.1^+^*i*NKT cells. These data suggest that DGKα and DGKζ may regulate the PKCθ/IKKβ/NF-κB axis during early *i*NKT cell development and the RasGRP1/Ras pathway during late *i*NKT cell development. However, further study using genetic and pharmacologic approaches is required to determine if increased PKCθ and RasGRP1 activation is causal for the observed defect in *i*NKT cell development with DGKα and DGKζ deletion.

## 8. Therapeutic Implications

The two most highly expressed DGKs in T cells, DGKα and DGKζ, are clearly involved in regulating many processes: T cell selection, *i*NKT cell development, T cell activation, T cell anergy, T cell responses to pathogen, and T cell anti-tumor responses. In a general sense, the attenuation and termination of TCR-mediated DAG signaling by DGKα and DGKζ is common to all these processes. As DAG has numerous downstream targets, one would predict that potential therapies to modulate DGK function would be most useful in areas where broad manipulation of TCR signaling is desirable, such as in suppressing autoimmunity or enhancing immune responses to cancer. However, one hurdle in targeting DGKα and DGKζ to modulate T cell function is that these isoforms are expressed endogenously in numerous tissues. In fact, based on EST data, DGKα and DGKζ are the most commonly tissue-expressed DGK isoforms of all ten DGKs [28]. In other tissues, DGKα and DGKζ regulate diverse processes, such as neurite outgrowth, leptin signaling, cardiac remodeling, and cancer cell migration and invasion [5,28]. DGKα- and DGKζ-modulating therapies may therefore be most useful in settings in which T cells can be specifically targeted, such as approaches using adoptive and autologous T cell transfer immunotherapies. Recently, autologous T cell transfer of chimeric antigen receptor (CAR)-transduced T cells was used successfully to treat patients with chronic lymphoid leukemia (CLL) [120,121]. CARs consist of a high affinity antigen-binding extracellular domain, a transmembrane domain, and an intracellular signaling domain [122]. The generation of productive signaling from CARs allows appropriate cellular activation, cytotoxicity, and persistence to specific tumor antigens, and how to induce productive signaling is a topic of much research [122]. DGKα and DGKζ could be attractive targets for augmenting the efficacy of CAR-expressing T cells, as these molecules modulate T cell signaling, activation, and anergy. Much of what is known about DGKα and DGKζ suggests that the overlap in function between these enzymes is considerable. Given the clear structural differences between DGKα and DGKζ, however, as yet uncovered isoform-specific functions probably exist. Targeting of therapies to modulate DGK function therefore would be aided by a more complete understanding of what these isoform-specific functions are and how these separate functions are regulated by other signaling events.

## 9. Conclusions and Future Directions

DGKs play a key role in regulating DAG, a crucial second messenger of TCR signaling. DGKα and DGKζ, the two most highly expressed isoforms of DGK in T cells, terminate DAG signaling and act to suppress TCR signaling. These molecules are highly regulated through transcriptional, post-translational and localization mechanisms by molecules important for TCR signaling. At the same time, DGKα and DGKζ control T cell function during activation and anergy, responses to pathogen and tumor, and the development of *i*NKT and T cells. Linking the regulation of DGKs with their function in T cells is an exciting and important area of future investigation.

Indeed, understanding the link between regulation of DGKs and their functions could be important for the targeting of these molecules for therapies. In addition, a more clear understanding of specific and selective roles of DGKα and DGKζ in T cells could aid the development of drugs targeting DGKs. Especially with the addition of adoptive and autologous T cell transfer therapies to our disease treatment repertoire, the future is bright for translating our understanding of DGK function to helping patients.
